# The Effect of Metformin on Polycystic Ovary Syndrome in Overweight Women: A Systematic Review and Meta-Analysis of Randomized Controlled Trials

**DOI:** 10.1155/2020/5150684

**Published:** 2020-09-16

**Authors:** Yuanyuan Guan, Dongjun Wang, Huaien Bu, Tieniu Zhao, Hongwu Wang

**Affiliations:** ^1^Graduate School, Tianjin University of Traditional Chinese Medicine, Tianjin 301617, China; ^2^School of Health Sciences and Engineering, Tianjin University of Traditional Chinese Medicine, Tianjin 301617, China

## Abstract

**Objective:**

Metformin is an important component of PCOS treatment. At present, the effect of metformin in overweight women with PCOS has not been evaluated. Therefore, we conducted a systematic review to assess the effects of metformin in overweight women with PCOS and to analyze the effects of metformin in overweight women with PCOS.

**Methods:**

We searched the PubMed, Cochrane Library, Embase, CNKI, VIP, and Wanfang databases for studies published before March 2020. Randomized controlled trials were identified to study the effects of metformin in overweight women with PCOS. Data from studies including body mass index (BMI), waist circumference (WC), follicle-stimulating hormone (FSH), homeostasis model assessment of insulin resistance (HOMA-IR), luteinizing hormone (LH), sex hormone-binding globulin (SHBG), high-density lipoprotein (HDL) cholesterol, low-density lipoprotein (LDL) cholesterol, total cholesterol (TC), triglycerides (TG), fasting blood glucose (FBG), fasting insulin, testosterone, and androstenedione were pooled. Qualified trials were selected, and methodological quality was strictly assessed. Two reviewers chose the studies independently of each other.

**Results:**

Twelve trials were included. The intervention group and the control group had significant differences in the changes in body mass index (BMI) (WMD = −1.25, 95% CI (−1.60, −0.91), *p* < 0.00001) and waist circumference (WC) (WMD = −1.41, 95% CI (−2.46, −0.37), *p*=0.008) after metformin. The comprehensive results show that, in all studies, overweight women with polycystic ovary syndrome treated with metformin had significantly improved endocrine and metabolic indicators, including testosterone, follicle-stimulating hormone, luteinizing hormone, and low-density lipoprotein cholesterol. However, metformin did not regulate the secretion indexes of fasting insulin, homeostasis model assessment of insulin resistance, sex hormone-binding globulin, high-density lipoprotein cholesterol, total cholesterol, triglycerides, fasting blood glucose, and androstenedione.

**Conclusions:**

Compared with control interventions, metformin appears to be an effective intervention for overweight women with PCOS.

## 1. Introduction

Polycystic ovary syndrome (PCOS) is a common gynaecological endocrine disease in women of childbearing age [1]. PCOS is characterized by excessive androgens, persistent anovulation, infertility, and metabolic disorders [2]. The morbidity rate is 6% to 15% among women during the childbearing period, and to date, the cause is not completely clear. Extensive clinical and epidemiological data show that approximately 50% of PCOS patients are overweight or obese [3]. Overweight women with PCOS suffer more severe endocrine and metabolic disorders than nonoverweight patients [4]. Studies have found that being overweight enhances insulin secretion but weakens the metabolism of insulin secretion in the liver, skeletal muscle, and fat. In addition to impaired insulin responsiveness of adipocytes, being overweight may also cause lipodystrophy and insulin resistance by reducing the expression of lipid droplet proteins in adipocytes [5, 6]. Karimi et al. [7] and Heshmati et al. [8] suggest that patients with polycystic ovary syndrome generally have insulin resistance and elevated serum insulin and abnormal lipoprotein metabolism. Studies have shown that overweight women with PCOS have a higher risk of type 2 diabetes, hypertension, hyperlipidaemia, cardiovascular disease, and metabolic syndrome [9]. Metformin is a biguanide insulin sensitizer [10]. It does not affect insulin secretion but can improve insulin action [11]. It is a first-line drug for the treatment of type 2 diabetes (T2 DM) [12]. Its mechanism of action is to reduce blood lipid levels, reduce liver glucose production, stimulate the liver and skeletal muscles to perform insulin-mediated glucose uptake, and reduce the utilization of gluconeogenic substrates [13]. Obese women with PCOS exhibit metabolic characteristics similar to those with T2 DM in terms of insulin resistance and hyperinsulinemia [14]. Since 1994, metformin has been used as an insulin sensitizer for the treatment of polycystic ovary syndrome [15]. Studies have shown that metformin can not only improve endocrine disorders in patients with PCOS but also regulate ovarian function and even reduce the weight of overweight women with PCOS [16]. Heidari et al. [17] believe that metformin can improve endothelial function and endothelial dysfunction in women with PCOS, but it has limited effects in improving glucose metabolism and dyslipidemia. From the current research status, the therapeutic effect of metformin on PCOS patients is still controversial, especially for overweight PCOS patients. In this study, a meta-analysis was performed to compare the metabolic regulatory effect of metformin in overweight women with PCOS.

## 2. Methods

### 2.1. Research Strategy

This meta-analysis was planned, conducted, and reported according to the Preferred Reporting Items for Systematic Reviews and Meta-Analyses (PRISMA) recommendations. We searched the PubMed, Cochrane Library, Embase, CNKI, VIP, and Wanfang databases for studies published before March 2020. Search terms including free terms and Medical Subject Heading terms (MeSH). The search terms were (“Metformin” OR “Metformin Hydrochloride” OR “Hydrochloride Metformin” OR “Dimethylbiguanidine”) AND (“polycystic ovary syndrome” OR “Stein-Leventhal Syndrome”) and randomized controlled trials (RCTs). Additionally, the reference lists of retrieved publications were also reviewed to identify relevant papers that might be missed during electronic database search. Two independent reviewers selected and screened all results and, in cases where they disagreed, a third reviewer was asked for advice. The review applied the PRISMA statement guidelines for reporting systematic reviews and meta-analyses [18].

### 2.2. Eligibility Criteria

The inclusion criteria for this systematic review were as follows: (1) the study design was a randomized controlled trial (RCT) related to the effect of metformin on PCOS in overweight women; (2) recruit humans as subjects, and the subject's BMI >25 kg/m^2^; (3) metformin was listed as the main intervention in the experimental group and compared with the nonintervention control status; and (4) at least one metabolic parameter was reported, and data including the mean and standard deviation of each group at baseline and postintervention as well as the number of participants in each group were available. The exclusion criteria were as follows: (1) duplicate publications; (2) nonintervention designs (such as case-control studies, cohort studies, cross-sectional studies, case reports and experiences, theory research, and reviews); and (3) nonclinical tests and animal experiments.

### 2.3. Data Extraction

Two review authors independently screened the literature using the predetermined inclusion criteria and extracted data from the trials. The following information was extracted: participant characteristics, intervention and outcome data, adverse effects, and methodological quality. We resolved any disagreements about the extracted data from the included studies by consensus and consulted a third review author if disagreements persisted.

### 2.4. Risk of Bias Assessment

The risk of study bias was assessed using the Cochrane Handbook for Systematic Reviews. The risk of bias was evaluated with regard to the following aspects: generation of random sequences, allocation of hidden methods, application of the blinding method, incomplete results, selective reporting of results, and other bias. Funnel diagrams were used to detect publication bias.

### 2.5. Statistical Analysis

#### 2.5.1. Extracting and Merging of Data

The Cochrane Collaboration's Review Manager 5.3 software was used to extract the relevant dichotomous or continuous data from the literature for analysis. Risk ratios (RRs) were calculated for dichotomous data, whereas the mean differences (MDs) and standard deviations (SDs) were calculated for continuous variables. The corresponding 95% confidence intervals (CIs) and forest plots were used in both cases. In our meta-analysis, we used SDs when the data had the same units. When they had different units, we performed a conversion. The chi-squared and *I*^2^ (inconsistency) tests were used to detect heterogeneity. A *p* value <.10 or *I*^2^ >50% indicated that there was significant heterogeneity. The fixed-effects model was used when *p* > 10 and *I*^2^ < 50%, and the random-effects model was used when *p* < 10 or *I*^2^ ≥ 50%.

#### 2.5.2. Data Conversion

The final values of body mass index (BMI), waist circumference (WC), follicle-stimulating hormone (FSH), homeostasis model assessment of insulin resistance (HOMA-IR), luteinizing hormone (LH), sex hormone-binding globulin (SHBG), high-density lipoprotein (HDL) cholesterol, low-density lipoprotein (LDL) cholesterol, total cholesterol (TC), triglycerides (TG), fasting blood glucose (FBG), fasting insulin, testosterone, and androstenedione were used as indicators to evaluate the efficacy of metformin in the intervention group and the control group. If the abovementioned metabolic indicators were not explicitly reported in the study, we calculated the mean value and SD of metabolic indicators with the following formulas:

1. If the number of samples (*n*) and the standard error (SE) were known, the SD was calculated as

(1) $$\begin{array}{c} \text{SD}=\text{SE}\times \sqrt{n}. \end{array}$$

(2) Estimates of the SD were calculated if the number of samples (*n*), mean, and 95% CI [19–21] were known: “a” and “b” are the upper and lower confidence limits, respectively:

(2) $$\begin{array}{c} \text{SD}=\frac{a-\text{mean}}{1.96\sqrt{n}},\\ \text{SD}=\frac{\text{mean}-b}{1.96\sqrt{n}}. \end{array}$$

## 3. Results

### 3.1. Study Selection

**Figure 1:**
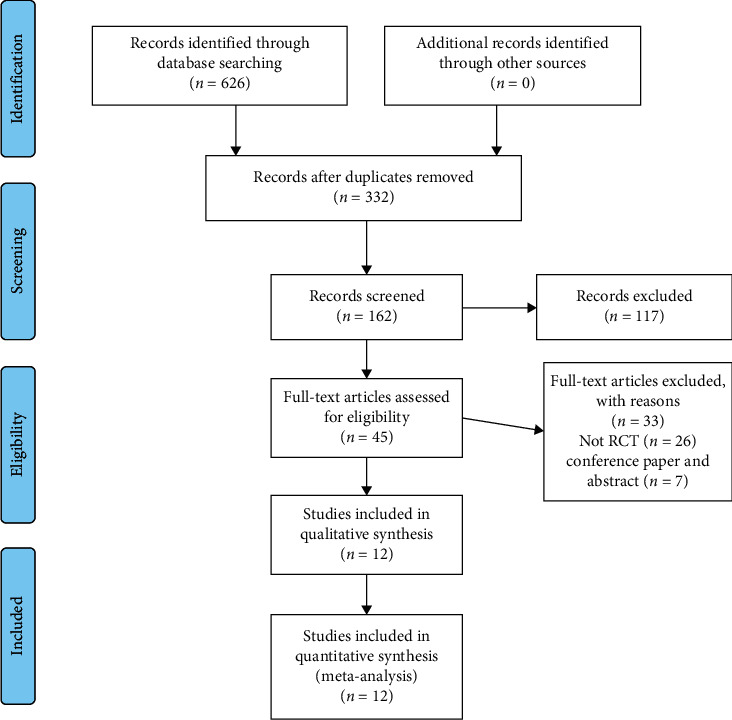
Study selection procedure according to the PRISMA statement.

A total of 626 study reports were screened, 294 of which were excluded because they were duplicate publications. After reading the titles and abstracts, an additional 170 articles were excluded, and 162 articles were retained. Among them, 117 articles did not meet the inclusion criteria, 15 studies were improperly compared, and in 18 studies, we could not extract the data. Finally, twelve RCTs with a total of 683 participants were included. The PRISMA flow diagram is shown in Figure 1.

### 3.2. Study Characteristics

The principal study characteristics are summarized in Table 1. Twelve studies were published between 2002 and 2019. A total of 683 participants were included. The number of participants in the individual studies ranged from 9 to 74. All of the included trials were single-center studies. The included studies came from different countries: United States [27], United Kingdom [30, 31], Iran [23, 28, 29], Brazil [22, 24], Italy [25, 27], India [32], and Turkey [33]. The duration of the intervention varied from 6 to 48 weeks. All participants had PCOS and a BMI >25 kg/m^2^. In the included studies, in the intervention group, the metformin intervention doses ranged from 750 mg to 2000 mg.

### 3.3. Quality Assessment

**Figure 2:**
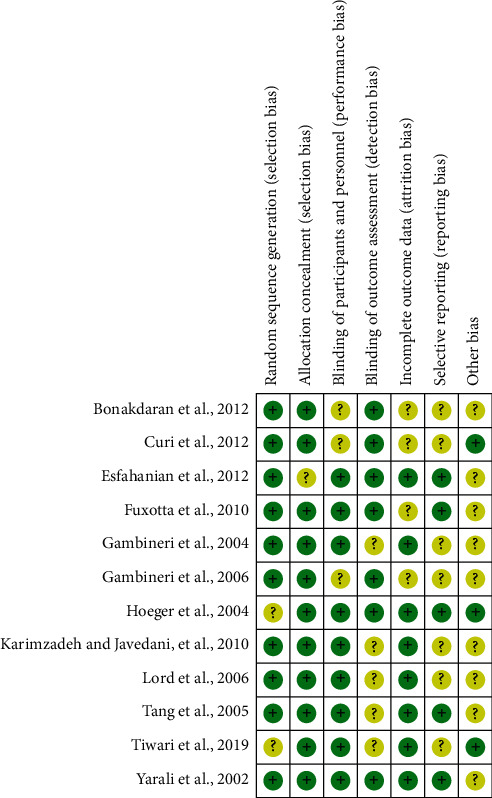
Risk of bias summary: review of authors' judgements about each risk of bias item for each included study.

Figure 2 provides an overview of the risk of bias for the included studies based on the tools provided by the Cochrane Manual. All included studies used a double-blind approach and reported dropouts. Most trials reported allocation concealment and random allocation but did not mention the specific method used. Five studies [25–27, 30, 31] reported automatic generation of random sequences by a computer, while two studies [23, 28] reported that they divided participants into an experimental group and a control group by using random number tables. Selective reporting was unbiased but without any description to evaluate the existence of other biases. All the included trials reported whether adverse events occurred.

### 3.4. Study Results

#### 3.4.1. BMI

**Figure 3:**
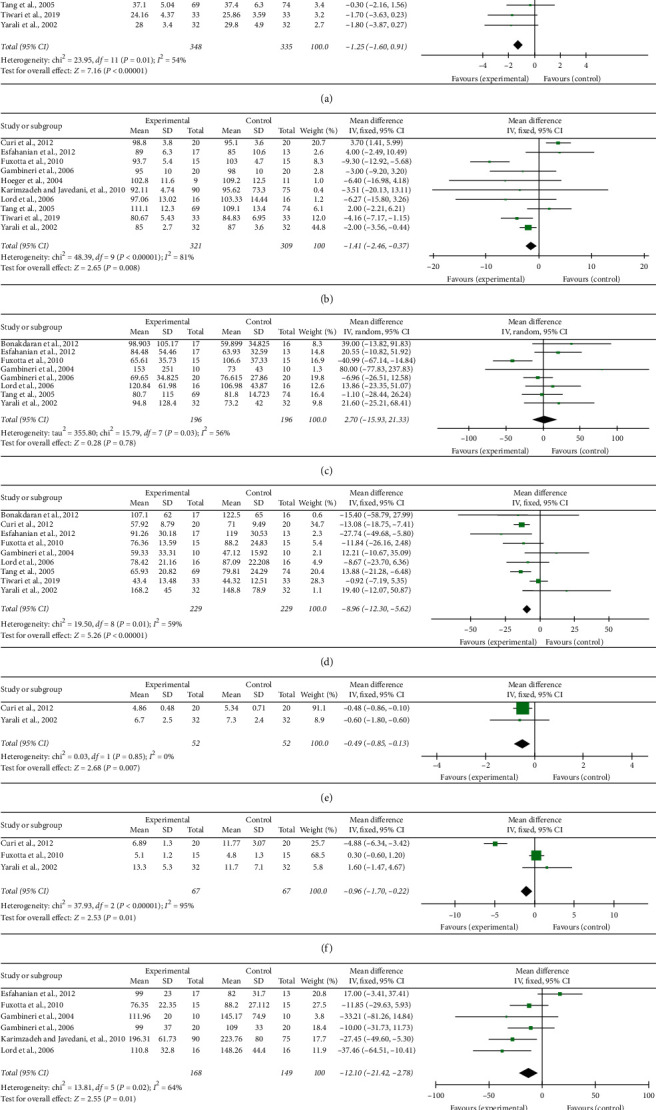
Effect of metformin on (a) body mass index; (b) waist circumference; (c) fasting insulin; (d) testosterone; (e) follicle-stimulating hormone; (f) luteinizing hormone; and (g) low-density lipoprotein.

Figure 3(a)shows the forest plots of the BMI analysis. The number of RCTs included was twelve. The combined results were statistically significant (WMD = −1.25, 95% CI (−1.60, −0.91), *p* < 0.00001). Compared with the control group, metformin had a positive effect on BMI in overweight women with PCOS. We used a fixed-effects model for the quantitative BMI data and showed low heterogeneity (*I*^2^ = 54%, *p*=0.01).

#### 3.4.2. Waist Circumference

In terms of reducing waist circumference, there was a significant difference between the metformin group and the control group (WMD = −1.41, 95% CI (−2.46, −0.37), *p*=0.008) (Figure 3(b)). There was substantial heterogeneity among the included studies (*I*^2^ = 81%, *p* < 0.00001).

#### 3.4.3. Fasting Insulin

The combined results of eight studies showed that overweight women with PCOS in the metformin group did not have significantly reduced fasting insulin (WMD = 2.70, 95% CI (−15.95, 21.33), *p*=0.78); these studies had low heterogeneity (I^2^ = 56%, *p*=0.03) (Figure 3(c)).

#### 3.4.4. Testosterone

Nine included trials including 458 participants [26, 31–33] reported data on changes in testosterone following metformin use. There was some heterogeneity in testosterone between overweight women with PCOS participating in the metformin intervention and those in the control group (*I*^2^ = 59%, *p*=0.01). Compared to the control group, the testosterone levels in the metformin group were reduced, and there were significant differences (WMD = −8.96, 95% CI (−12.30, −5.62), *p* < 0.00001) (Figure 3(d)).

#### 3.4.5. Study on the Comprehensive Efficacy of Metformin

Studies investigated the effects of metformin on ten outcomes (FSH (follicle-stimulating hormone), HOMA-IR (homeostasis model assessment of insulin resistance), LH (luteinizing hormone), SHBG (sex hormone-binding globulin), HDL (high-density lipoprotein) cholesterol, LDL (low-density lipoprotein) cholesterol, TC (total plasma cholesterol), TG (triglycerides), FBG (fasting blood glucose), and androstenedione).

**Figure 4:**
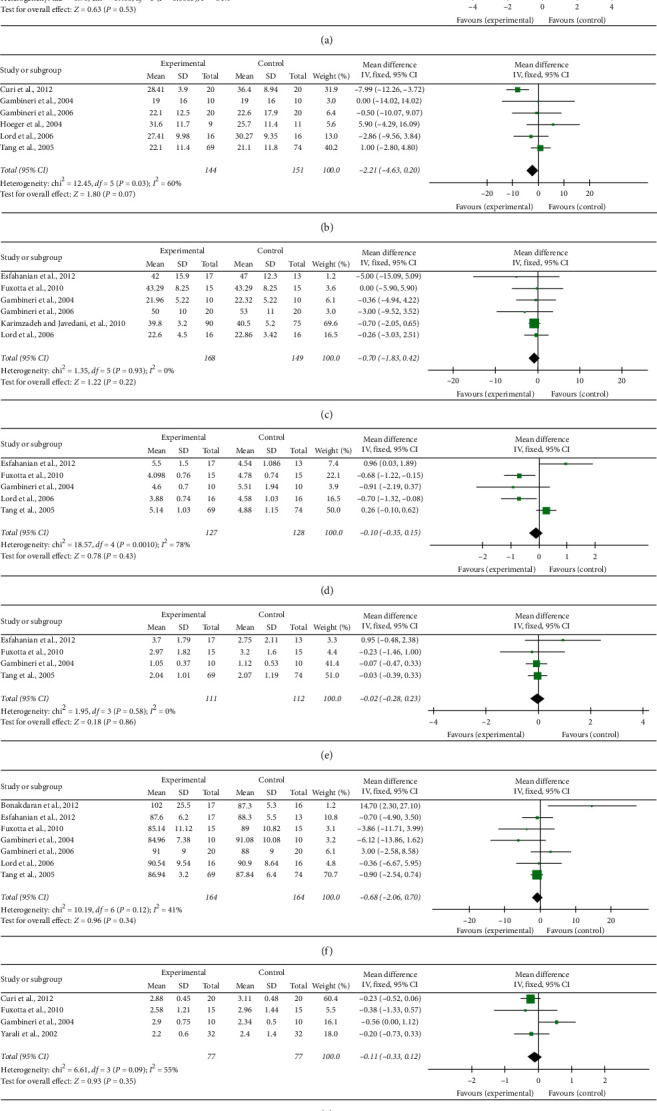
Effect of metformin on (a) homeostasis model assessment of insulin resistance; (b) sex hormone-binding globulin; (c) high-density lipoprotein; (d) total plasma cholesterol; (e) triglycerides; (f) fasting blood glucose; and (g) androstenedione.

The synthesized results showed positive effects of metformin on FSH (WMD = −0.49, 95% CI −0.85 to −0.13, *p*=0.007, *I*^2^ = 0%; Figure 3(e)), LH (WMD = −0.96, 95% CI −0.17 to −0.22, *p*=0.01, *I*^2^ = 95%; Figure 3(f)), and LDL cholesterol (WMD = −12.10, 95% CI 0.22 to 1.00, *p*=0.01, *I*^2^ = 64%; Figure 3(g)). There was not significant difference in HOMA-IR (SMD = 0.29, 95% CI −0.61 to 1.18, *p*=0.53, *I*^2^ = 84%; Figure 4(a)), SHBG (WMD = −2.21, 95% CI −4.63 to 0.20, *p*=0.007, I^2^ = 60%; Figure 4(b)), HDL cholesterol (WMD = −0.70, 95% CI −1.83 to 0.42, *p*=0.22, I^2^ = 0%; Figure 4(c)), TC (WMD = −0.10, 95% CI −0.35 to 0.15, *p*=0.43, *I*^2^ = 78%; Figure 4(d)), TG (WMD = −0.02, 95% CI −0.28 to 0.23, *p*=0.86, *I*^2^ = 0%; Figure 4(e)), FBG (WMD = −0.68, 95% CI −2.06 to 0.07, *p*=0.34, *I*^2^ = 41%; Figure 4(f)), or androstenedione (WMD = −0.11, 95% CI −0.33 to 0.12, *p*=0.35, *I*^2^ = 55%; Figure 4(g)), between overweight women with PCOS who received a metformin intervention and those in the control group.

### 3.5. Publication Bias

**Figure 5:**
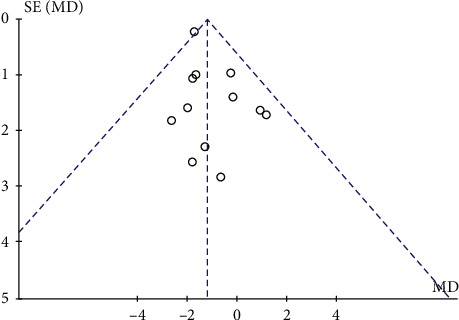
Funnel plot of publication bias.

The publication bias of the twelve RCTs was evaluated with a funnel plot.  Figure 5 shows that the publication bias across the studies was small.

## 4. Discussion

Polycystic ovary syndrome (polycystic ovary syndrome, PCOS) is a gynaecological endocrine disorder commonly seen in women of reproductive age and has highly heterogeneous clinical manifestations [34]. Approximately 70% of PCOS patients are overweight or obese, and PCOS may be related to genetic, environmental factors including diet, lifestyle, and hormone levels [35]. Obesity as a risk factor often causes female diseases such as breast cancer [36]. Studies have found that, with increases in weight, abnormal genes such as the Wnt signalling pathway, oxidative stress, and inflammation in adipose tissue of PCOS patients are abnormal [37], suggesting that obesity participates in the pathogenesis of PCOS [38], triggers metabolic and reproductive disorders, and may also cause glycolipid metabolism, hyperandrogenaemia, menstrual disorders, infertility, and comorbidities related to polycystic ovary syndrome [39]. Furthermore, we also noticed that many features and complications of polycystic ovary syndrome (PCOS) can trigger oxidative stress and increase insulin resistance index [40, 41]. Obese women with PCOS show lower ISOGTT and higher LH to stimulate androgen secretion, triggering insulin resistance and excessive androgens [42]. Current evidence-based guidelines recommend that overweight women with PCOS use metformin to control their weight and endocrine and metabolic disorders [43]. As the most widely used insulin sensitizer for PCOS, metformin can reduce liver glucose production, inhibit gluconeogenesis and adipogenesis, and improve peripheral tissue insulin sensitivity [44]. In addition, a large number of studies have shown that metformin can not only reduce weight and metabolic disorders but also correct menstrual patterns, restore ovulation, and even allow conception [45, 46]. Furthermore, in previous systematic reviews, the specific therapeutic effect of metformin on metabolic indicators in overweight women with PCOS has not been evaluated. Through quantitative synthesis, we found that as a drug that regulates the metabolism of overweight women with PCOS, metformin seems to have a partial effect, can reduce BMI and WC, and can reduce testosterone, FSH, LH, and LDL cholesterol.

### 4.1. Summary of the Main Results

Combined with our research results, we found that taking metformin can reduce body mass index, waist circumference, FSH, LH, LDL cholesterol, and testosterone levels in overweight women with PCOS. However, there was no improvement in fasting insulin levels, HOMA-IR, LDL cholesterol levels, HDL cholesterol levels, SHBG levels, FBG levels, androstenedione levels, TC levels, or TG levels. Our current results suggest that metformin may be the most effective intervention for PCOS in overweight women [47]. The results show that the improvement of body mass index, waist circumference, and LDL cholesterol may be the direct regulation effect of metformin on the production of ovarian steroids [11, 48]. Our research results found that metformin has a lowering effect on FSH in overweight PCOS patients. It can be considered as abnormal gonadotrophic secretion in women with overweight PCOS, which makes FSH in an abnormal secretion stage [49]. The antireproductive effect of metformin helps correct this phenomenon [50]. The production of polycystic ovary syndrome is directly related to the abnormality of insulin. Insulin resistance will cause hyperinsulinemia, which directly affects the role of ovarian receptors, inhibits insulin-binding protein and sex hormone-binding protein, while freeing testosterone and increasing ovarian androgens. Therefore, metformin is used to regulate insulin secretion and achieve the purpose of effectively improving polycystic ovary syndrome. This finding is consistent with international guidelines for the management of overweight and diseased adults and overweight people. Most approved weight management drugs are contraindicated in women of reproductive age, but metformin has fewer side effects, is safer, and is recommended for use in PCOS treatment [51]. Clinical studies of overweight women with PCOS have found that endocrine disorders can lead to infertility [52]. This study found that metformin has a certain regulatory effect on PCOS sex hormones in overweight women, can promote luteinizing hormone secretion, achieve ovulation, and improve the menstrual cycle of patients. In addition, it has the function of regulating follicle-stimulating hormone secretion. Some studies have suggested that the abnormal state of ovarian ultrasound detection in patients with polycystic ovary syndrome is closely related to testosterone levels [53]. Studies have also confirmed that reduced testosterone levels can effectively improve the hyperandrogenaemia of PCOS in overweight women and improve clinical symptoms such as excess hair, black acanthosis, and acne [54].

### 4.2. Limitations

This study has several limitations. First, in some cases, we had to calculate and transform data rather than data being provided directly. Second, the study distribution between the twelve RCTs was included, which may affect the meta-analysis results. The results of the included studies showed significant differences, which may be due to the different metformin doses, durations, center settings, and selected populations of different treatment programs. We performed a sensitivity analysis of the included RCTs and found that two studies may be a source of most of the heterogeneity. In both studies, different laboratory tests were used, which may have an impact on the comprehensive measurement results. In addition, language, publication bias, and not being registered with PROSPERO limit our research. Finally, this review only included randomized controlled trials. In the future, there is a need for a greater diversity of research, such as cooperation between multiple centres, more rigorous clinical reports, and prospective studies.

### 4.3. Clinical Implications

We summarize the current research status of metformin in overweight women with PCOS and provide data to support future PCOS clinical trials. Although this study shows that metformin can effectively regulate the levels of BMI and physiological function indicators in overweight PCOS women, more clinical studies are needed in the future to prove that effective prevention can reduce the occurrence of complications (such as hypertension and stroke) [55, 56]. This result should be interpreted with caution due to the insufficient quality of current evidence research methods and the observed clinical heterogeneity. In future studies, further attention should be paid to the effects of metformin dosage and intervention time in overweight women with PCOS. In conclusion, this study found that metformin has a certain regulatory effect on the relevant physiological indicators of overweight women with PCOS.

## 5. Conclusion

Compared with control interventions, metformin appears to be an effective intervention for overweight women with PCOS. We have to admit that this study may have some serious limitations. Different treatment options, doses, duration, and enrolment of different populations may have led to obvious heterogeneity, and we need to interpret the results carefully. More RCTs with a rigorous research design are needed to determine the efficacy of metformin in treating PCOS patients, to evaluate the risk factors in overweight women, and to apply metformin in interventions for nonoverweight PCOS patients to prevent or treat the occurrence of PCOS and its complications.

## Figures and Tables

**Table 1:** Systematic review of randomized controlled trials evaluating the effects of metformin in overweight women with polycystic ovary syndrome.

Author	Sample size (T/C)	Mean age (T/C)	BMI (T/C)	Nation	Intervention	Control	Metformin intervention			Outcome measured
							Frequency (per day)	Dose (mg)	Duration (week)	
Curi et al. [22]	20/20	24.6 ± 1.3/26.3 ± 1.4	31.1 ± 1.5/31.8 ± 1.6	Brazil	Metformin	Usual care controls + placebo treatment	2	850 mg	24	①②③④⑤⑥⑬⑭

Esfahanian et al. [23]	17/13	21.9 ± 9.3/20 ± 4.6	31.1 ± 3.3/34.1 ± 5.4	Iran	Metformin	Usual care controls + hypocaloric diet	—	1000 mg/day gradually to 2000 mg/day	12	①②⑦⑧⑨⑩⑪⑫⑬

Otta et al. [24]	15/15	25.47 ± 4.82/24.7 ± 3.46	32.4 + 6.7/35.6 + 4.98	Brazil	Metformin	Usual care controls + placebo treatment	2	750 mg	16	①②④⑤⑦⑧⑨⑩⑪⑫⑬⑭

Gambineri et al. [25]	10/10	26·1 ± 4·5/27·1 ± 3·6	37·0 ± 5·9/37·6 ± 4·1	Italy	Metformin	Usual care controls + placebo treatment	2	850 mg	24	①⑥⑦⑧⑨⑩⑪⑫⑬⑭

Gambineri et al. [26]	20/20	2 8 ± 8/26 ± 5	35 ± 4/37 ± 5	Italy	Metformin	Usual care controls + placebo treatment	2	850 mg	48	①②⑥⑦⑧⑪⑫

Hoeger et al. [27]	9/11	29.5 ± 6.4/27.1 ± 4.5	37.1 ± 4.9/37.1 ± 4.6	United States	Metformin	Usual care controls + placebo treatment	2	850 mg	48	①②⑥

Bonakdaran et al. [28]	17/16	25.9 ± 4.5/25.2 ± 7.9	28.2 ± 5.03/25.3 ± 5.1	Iran	Metformin	Usual care controls + placebo treatment	2	1000 mg	12	①④⑪⑫⑬

Karimzadeh Javedani [29]	90/75	27.33 ± 2.34/27.48 ± 2.69	27.17 ± 1.73/27.92 ± 1.05	Iran	Metformin	Usual care controls + placebo treatment	2	1500 mg	24	①②⑦⑧

Lord et al. [30]	16/16	27.76 ± 4.89/30.63 ± 4.84	33.74 ± 6.74/36.37 ± 7.46	United Kingdom	Metformin	Usual care controls + placebo treatment	3	500 mg	12	①②④⑥⑦⑧⑨⑩⑪⑫⑬

Tang et al. [31]	69/74	29.7 ± 3.7/29.8 ± 3.8	37.6 ± 5.0/38.9 ± 9.5	United Kingdom	Metformin	Usual care controls + placebo treatment	2	850 mg	24	①②⑥⑨⑩⑪⑫⑬

Tiwari et al. [32]	33/33	24.33 ± 3.89/24.46 ± 4.76	25.23 ± 4.64/26.32 ± 3.68	India	Metformin	Usual care controls + fixed exercise	2	850 mg	24	①②⑬

Yarali et al. [33]	32/32	29.7 ± 5.6/28.4 ± 5.1	28.6 ± 4.0/29.6 ± 4.8	Turkey	Metformin _+_ rFSH	Usual care controls + placebo treatment	2	850 mg	6	①②③⑤⑫⑬⑭

Months were converted to weeks by using 1 month = 4 weeks; years were converted by using 1 year = 52 weeks. BMI: body mass index; WC: waist circumference; FSH: follicle-stimulating hormone; HOMA-IR: homeostasis model assessment of insulin resistance; LH: luteinizing hormone; SHBG: sex hormone-binding globulin; HDL: high-density lipoprotein; LDL: low-density lipoprotein; TC: total cholesterol; TG: triglycerides; and FBG: fasting blood glucose. ① BMI; ②WC; ③ FSH; ④ HOMA-IR; ⑤ LH; ⑥SHBG; ⑦ HDL; ⑧ LDL; ⑨ TC; ⑩ TG; ⑪ FBG; ⑫ fasting insulin;⑬ testosterone; and ⑭ androstenedione.

## Data Availability

All the data in this paper support the results of this study.

## Abbreviations

CI: Confidence interval
CNKI: China National Knowledge Infrastructure
MD: Mean difference
PRISMA: Preferred Reporting Items Systematic Reviews and Meta-Analyses
PubMed: National Library of Medicine
RCT: Randomized controlled trial
RR: Risk ratio
SD: Standard deviation
SE: Standard error
VIP: VIP Database for Chinese Technical Periodicals
WMD: Weighted mean difference
PCOS: Polycystic ovary syndrome
BMI: Body mass index
WC: Waist circumference
FSH: Follicle-stimulating hormone
HOMA-IR: Homeostasis model assessment of insulin resistance
LH: Luteinizing hormone
SHBG: Sex hormone-binding globulin
TG: Triglycerides
TC: Total cholesterol
HDL: High-density lipoprotein
LDL: Low-density lipoprotein
FBS: Fasting blood glucose.

## Supplementary Materials

Supplementary Materials PRISMA checklist used in the study.
